# Calculating the optimal surveillance for head and neck paraganglioma in *SDHB*-mutation carriers

**DOI:** 10.1007/s10689-016-9923-3

**Published:** 2016-08-29

**Authors:** Karin Eijkelenkamp, Thamara E. Osinga, Mirjam M. de Jong, Wim J. Sluiter, Robin P. F. Dullaart, Thera P. Links, Michiel N. Kerstens, Anouk N. A. van der Horst-Schrivers

**Affiliations:** 1 Department of Endocrinology and Metabolic Diseases, University Medical Center Groningen, University of Groningen, Hanzeplein 1, 9700 RB Groningen, The Netherlands; 2 Department of Clinical Genetics, University Medical Center Groningen, University of Groningen, Groningen, The Netherlands

**Keywords:** Paraganglioma, Pheochromocytoma, *SDHB* mutation carriers, Surveillance

## Abstract

Germline mutations of the gene encoding succinate dehydrogenase subunit B (*SDHB*) predispose to head-and-neck-paraganglioma (HNPGL), sympathetic PGL, pheochromocytoma and renal cell carcinoma for which regular surveillance is required. *SDHB*-associated tumors harbor germline and somatic mutations, consistent with Knudson’s two-hit hypothesis. To assess the penetrance and optimal surveillance for different manifestations of *SDHB* mutation carriers. This study included all *SDHB* mutation carriers who were followed at the Department of Endocrinology at the University Medical Center of Groningen. Kaplan–Meier curves were used to assess the penetrance. Poisson process was used to assess the optimal age to start surveillance and intervals. Ninety-one *SDHB*-mutation carriers (38 men and 53 women) were included. Twenty-seven mutation carriers (30 %) had manifestations, with an overall penetrance 35 % at the age of 60 years. We calculated that optimal surveillance for HNPGL could start from an age of 27 years with an interval of 3.2 years. This study underscores the relatively low penetrance of disease in *SDHB* mutation carriers. Use of the Poisson approach provides a more accurate estimation of the age to initiate surveillance and length of intervals for HNPGL. These results may give rise to reconsider the current guidelines regarding the screening of these mutation carriers.

## Introduction

The succinate dehydrogenase subunit B (*SDHB*) gene is one of the 15 [1] susceptibility genes that have been linked to familial paraganglioma (PGL) and pheochromocytoma (PCC) [2]. Germline mutations in this gene predispose to head and neck paragangliomas (HNPGLs), sympathetic PGLs and PCCs. HNPGLs are mainly nonsecretory tumors of the parasympathetic paraganglia in the head and neck region, whereas sympathetic PGLs and PCCs are known for overproduction of catecholamines. Because of increased risk of developing HNPGLs, sympathetic PGLs and PCC, *SDHB* mutation carriers require regular surveillance, which consists of determination of plasma and/or urine metanephrines and performance of magnetic resonance imaging (MRI) at regular intervals (Table 1) [3–8].

**Table 1:** Recommendations for surveillance of *SDHB* mutation carriers

	Age to start surveillance (years)	Surveillance program		
		Biochemical tests	Anatomical imaging	Functional imaging
Kirmani et al. [4]	10^a^	Annual	CT/MRI of skull base and neck every 2 years, MRI of TAP every 4 years	^123^I-MIBG every 4 years
Benn et al. [8]	10	Annual	CT and/or MRI neck and TAP every 2 years	Consider ^18^F-DOPA-PET
Neumann et al. [6]	Not reported	Annual	Annual MRI of neck and TAP	Consider ^18^F-DOPA-PET
Srirangalingam et al. [7]	5	Annual	Annual MRI of neck and TAP	
Dutch guideline [3]	18	Annual	MRI of TAP every 2 years MRI of head and neck every 3 years	
Taïeb et al. [5]	Not reported	Annual	MRI of head and neck every 3 years	PET should be discussed on individual cases

*SDHB* succinate dehydrogenase sununit B, *CT* computed tomography, *MRI* magnetic resonance imaging, ^*123*^ *I-MIBG* ^123^metaiodobenzylguanine, ^*18*^ *FDOPA-PET* 6-[18F]-fluoro-L-3,4-dihydroxyphenylalanine positron emission tomography, *TAP* thoracic-abdominal-pelvic region

^a^Or at least 10 years before earliest age at diagnosis in the family

According to the current literature, the mean age upon diagnosis of a manifestation in *SDHB* mutation carriers is 30–35 years [9]. Various surveys report considerable differences in disease penetrance ranging from 8 to 55 % at the age of 40 years [7–12]. A mutation in the *SDHB* gene is also associated with a high risk of the development of malignant PGLs, varying from 17 to 71 % [7, 8, 12–14]. In addition, *SDHB* mutation carriers have been associated with an increased risk of developing other neoplasms, including renal cell carcinoma, gastrointestinal stromal tumors (GISTs) and papillary thyroid cancer [9]. Until today, no international consensus has been developed regarding the optimal mode of surveillance for *SDHB* mutation carriers. The Dutch national guideline advises to screen *SDHB* mutation carries as early as from the age of 18 years, including annual physical examination, biochemical testing for catecholamine excess (plasma/urinary metanephrines) and regular imaging of the head and neck and thoracic-abdominal-pelvic region [3]. These recommendations correspond to international guidelines, except for the age of starting surveillance and the subsequent intervals (Table 1).

*SDHB*-associated tumors harbor germline and somatic mutations, consistent with Knudson’s two-hit hypothesis [15]. This hypothesis states that the combination of an inactivating germline mutation as a first hit and somatic loss of function of the wild type allele as a second hit is essential for tumor development [16, 17].

Recently, Knudson’s two hit hypothesis has been used with von Hippel Lindau (*VHL*) mutation carriers to calculate time-to-detection of the first and subsequent VHL-related manifestations [18]. This model could also provide a rationale to define the age to start surveillance and subsequent surveillance intervals in patients with *SDHB* mutations. This would allow for evidence-based decisions as to the age to start surveillance in patients with a *SDHB* mutation and the length of the subsequent surveillance intervals. Therefore, the aim of this study was to assess the penetrance and optimal surveillance for different manifestation of *SDHB* mutation carriers, using a Poisson distribution model.

## Patients and methods

### Study population

All *SDHB* mutation carriers undergoing surveillance at the Endocrinology department of the University Medical Center Groningen (UMCG) between 2008 (establishment of guidelines for surveillance) and July 2015, were included in this study. The index patients of a *SDHB*-carrier family were also included when known at the UMCG.

Screening of *SDHB* mutation carriers was performed according to Dutch surveillance guidelines, consisting of physical examination and measurements of plasma/urinary metanephrines every year, MRI of the head and neck region every 3 years and MRI of the thoracic-abdominal-pelvic region every 2 years [3].

Demographics and clinical data including the type of mutation, tumor characteristics, symptoms, blood pressure, biochemistry, radiologic imaging records (original report) and treatment were collected from the medical charts. Manifestations were classified as either HNPGL (carotid-, jugular/tympanic- or vagal body), sympathetic PGL (thoracic, abdominal or pelvic PGL) or PCC The presence of a primary manifestation or metastases was confirmed histologically or based on a combination of persistently elevated plasma/urinary metanephrines (in case of a sympathetic PGL or PCC) and functional imaging including ^111^In-octreotide scintigraphy (octreoscan), ^123^I-metaiodobenzylguanidin (MIBG scintigraphy) and/or 6-[^18^F]-fluoro-L-3,4-dihydroxyphenylalanine (DOPA) positron emission tomography (PET) and/or anatomical imaging (Computed Tomography (CT) and/or MRI). Carriers who developed a manifestation were classified as disease “affected carriers”; i.e. either index patients or carriers who developed a manifestation detected during follow-up. Carriers were classified as “unaffected” when there was no evidence of a HNPGL, sympathetic PGL or PCC on the last performed imaging. Other neoplasms were also recorded when histological proven.

According to the Dutch Medical Research Involving Human Subjects Act, no further Institutional Review Board approval was required, because we used existing clinical data already collected for regular patient care. The identity of the subjects was protected by using unique codes which were only known to the principal investigator.

### Measurement of urinary and plasma metanephrines

Isotope-dilution mass spectrometry based measurements of urinary metanephrines [metanephrine (MN), normetanephrines (NMN) and 3-methoxytyramine (3-MT)] were used, with the following reference intervals: MN 33–99 µmol/mol creatinine, NMN 64-260 µmol/mol creatinine, 3-MT 45–197 µmol/mol creatinine. Plasma free metanephrines assays were performed with a High-Performance Liquid Chromatography tandem mass spectrometric technique (LC–MS/MS) with automated solid phase extraction sample preparation, as described by de Jong et al. [19]. Reference intervals for plasma free metanephrines were: MN 0.07–0.33 nmol/L, NMN 0.23–1.07 nmol/L, 3-MT <0.17 nmol/L. Elevated plasma/urinary metanephrines were defined as a value above the upper reference limit.

### Imaging

MRI scans of head and neck region were performed on a 1.5 Tesla scanner with 4 mm coronal and axial pre- and postcontrast T1-weighted sequences covering the posterior skull base and neck, as well as a dynamic contrast-enhanced MRA from aortic arch to skull base. MRI scans of thoracic-abdominal region were performed on a 1.5 Tesla scanner with coronal and transversal T1- and T2-weighted sequences without contrast.

Functional imaging (octreoscan, MIBG scintigraphy or DOPA-PET) was performed in all patients with sympathetic PGL and or PCC. It was also used in patients with HNPGL to specify the nature of the lesion found on MRI when the original report was inconclusive.

### Statistical analyses

Data are presented as mean ± standard deviation (SD) or as median with interquartile range [IQR] or range. Penetrance for the different manifestations of disease was determined using the reverse Kaplan–Meier method. For unaffected carriers the last follow-up date was defined as follows: date of the last negative imaging of head and neck region in case of HNPGL, and date of last negative imaging of thoracic-abdominal-pelvic region for sympathetic PGL and PCC.

Because including index patients is likely to bias towards a younger diagnosis and therefore affect the estimation of penetrance, we performed the statistical analysis both with and without the index patients. The log rank test was used to compare the penetrance between different genotypes.

The Poisson distribution model was used to calculate the probability of detection of a manifestation over time. As alluded to above, *SDHB*-related tumors are supposed to be consistent with Knudson’s two hit hypothesis, suggesting that the second hit (i.e. loss of the wild type allele and consequently to the development of a tumor) occurs at random [15, 17].

A linear regression analysis of this model was used to estimate the incidence κ (hit rate) of developing a manifestation. The time between the second hit and the detection of the tumor is called delay (δ). This time was assessed from the intercept of the natural logarithm (ln) of 1-cumulative proportion with the age axis. Only calculated cumulative proportions expected to follow the straight part of the line are used for the linear regression analyses. The initial part, set arbitrarily to below 3 %, will necessarily deviate from the straight line because of the variance in delay and is thus omitted in this analysis. The optimal age to start surveillance was calculated from the delay (δ) minus 2 * SD, the lower end of the 95 % confidence interval of the estimated delay. In this matter, a safe boundary was established with a negligible risk of a manifestation below this age of starting surveillance. The surveillance interval was defined by 0.1 * 1/κ, defining a 10 % change for the detection of a new tumor within this interval. This interval was calculated also for the proportions 5 and 1 %.

A two sided *P* value <0.05 was considered statistically significant. Statistical analyses were performed with PASW statistics (version 10; IBM/SPSS, Armonk, New York).

## Results

### Study population

Ninety-one carriers from 24 non-consanguineous families with a *SDHB* mutation were seen at the Department of Endocrinology between 2008 and 2015. Of these 91 carriers, 21 (23 %) were index patients. Twenty-seven carriers (30 %) were disease affected at the time of analysis. Eight different mutations in the *SDHB* gene were documented. Characteristics of mutation carriers are listed in Table 2.

**Table 2:** Patient characteristics of *SDHB* mutation carriers

	*SDHB* mutation carriers (*n* = 91)
Sex (male/female)	38/53
Index patients	21 (23 %)
Disease affected carriers	27 (30 %)
Mean age at first visit in years (±SD)	44 (±15)
Median duration of follow-up in years [IQR]	3.3 [2.2–4.5]
Median duration of follow-up in years [IQR], disease affected carriers	3.7 [2.3–11.3]
Median duration of follow-up in years [IQR], unaffected carriers	3.1 [2.1–4.1]
Manifestations of disease affected carriers	
HNPGL	19 (20 %)
Sympathetic PGL/PCC	9 (10 %)
Median age of diagnosis in years [IQR]	
HNPGL	45 [38–55]
Sympathetic PGL/PCC	19 [17–36]
Biochemical profiles of disease affected carriers^a^	
Silent tumors	16 (57 %)
Elevated normetanephrine	8 (29 %)
Elevated metanephrine	2 (7 %)
Elevated 3-MT	5 (18 %)
Malignant PGL	8 (9 %)
Mutations in *SDHB*-gene	
Deletion exon 3	44 (48 %)
c.654G>A	25 (27 %)
c.653G>A	14 (15 %)
c.649C>T	1 (2 %)
c.292T>C	4 (4 %)
c.268C>T	1 (1 %)
c.73?−200+	1 (1 %)
c.725G>A	1 (1 %)

*SDHB* succinate dehydrogenase subunit B, *HNPGL* head and neck paraganglioma, *PGL* paraganglioma, *PCC* pheochromocytoma, *SD* standard deviation, *IQR* interquartile range, *3-MT* 3-methoxytyramine

^a^The biochemical profile of one patient is missing

Median of follow-up of all carriers combined was 3.3 [IQR 2.2–4.5] years. Median follow-up for disease affected carriers (index patients and patients with manifestations detected during surveillance) and for unaffected carriers was 3.7 years [IQR 2.3–11.3] and 3.1 years [IQR 2.1–4.1], respectively. Three patients (all index patients) died during follow-up, due to malignant sympathetic PGLs. In three patients (3 %) no imaging of head and neck region was available at the end of follow-up (i.e. July 2015) and were therefore excluded for the calculation of the penetrance of HNPGL.

### Penetrance of HNPGLs

**Fig. 1:**
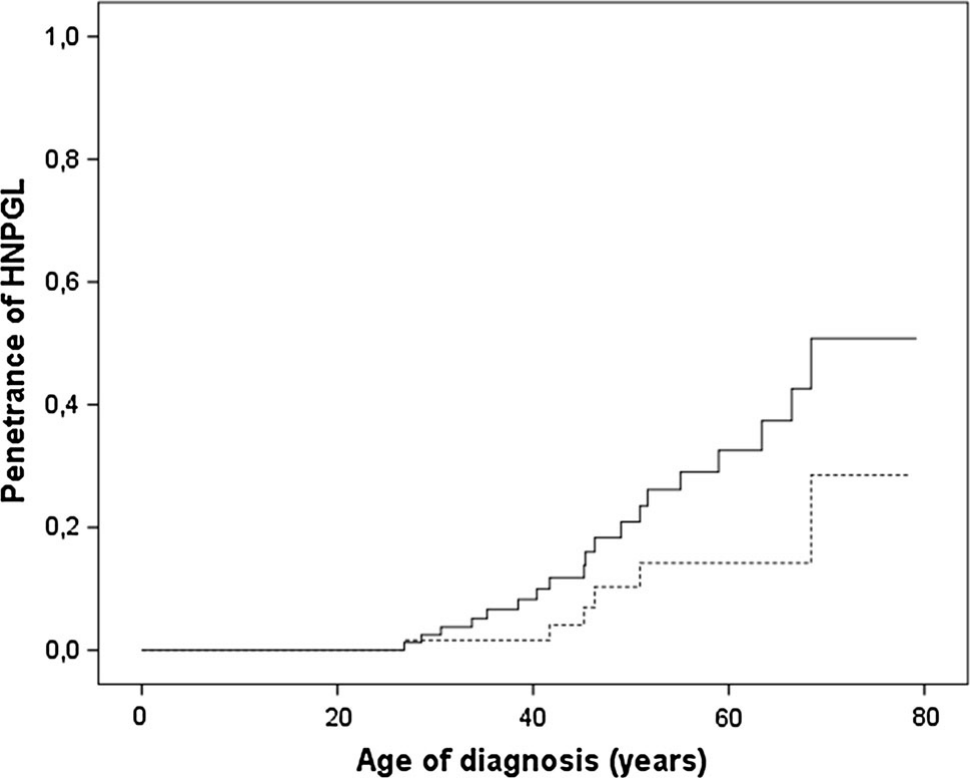
Age-related penetrance of succinate dehydrogenase subunit B mutation carriers with a head and neck paraganglioma (HNPGL), including index cases (*black line*), and excluding index cases (*dotted line*)

Nineteen *SDHB* mutation carriers were diagnosed with a HNPGL (*n* = 13 index patients, *n* = 6 detected during surveillance) (Table 2). These six HNPGL were detected by imaging during surveillance. Only one patient had an elevated plasma NMN level at the same time the imaging was performed. The penetrance of this manifestation, including all carriers, was 8 % at the age of 40 and 32 % at the age 60 years. When we excluded the index patients, the penetrance was 2 % at age 40 and 14 % at age 60 years (Fig. 1). No differences in penetrance were found between the most common mutations deletion exon 3 (*n* = 11) and c.653G>A (*n* = 5) (*P* = 0.787). The mutations c.654G>A (*n* = 1), c.292T>C (*n* = 1), c.725G>A (*n* = 1) were excluded for comparison due to the low number of patients. The median age at diagnosis of a HNPGL was 45 [IQR 38–55, range 26–68] years. Tumors were located in the carotid-(*n* = 10), jugular/tympanic-(*n* = 6) and vagal body (*n* = 3). Three patients had multiple HNPGLs. Two of these patients developed metastases, 31 and 22 years after the initial diagnosis of HNPGL.

### Penetrance of sympathetic PGLs and PCCs

Nine *SDHB* mutation carriers (all index patients), were diagnosed with a sympathetic PGL or PCC (Table 2). The mutations were: deletion exon 3 (*n* = 5), c.654G>A (*n* = 2), c.653G>A (*n* = 1), c.268G>A (*n* = 1). The penetrance of this manifestation was estimated to be 10 % at age 40 years and 10 % at age 60 years. Calculation of the penetrance after exclusion the index patients, or comparison of the penetrance according to the different types of mutation was not feasible due to the low number of patients. The median age at diagnosis was 19.7 years [IQR 17–36, range 11–66]. All sympathetic PGLs were located in the abdominal or pelvic region, mostly in the para-aortal region. One PGL was located in the organ of Zuckerkandl and one in the urinary bladder. Six out of the nine patients (67 %) with a sympathetic PGL or PCC developed metastases. Four patients presented with metastases, and an additional two patients developed metastases 4 and 39 years after the initial diagnosis of sympathetic PGL.

### Penetrance of all manifestations

**Fig. 2:**
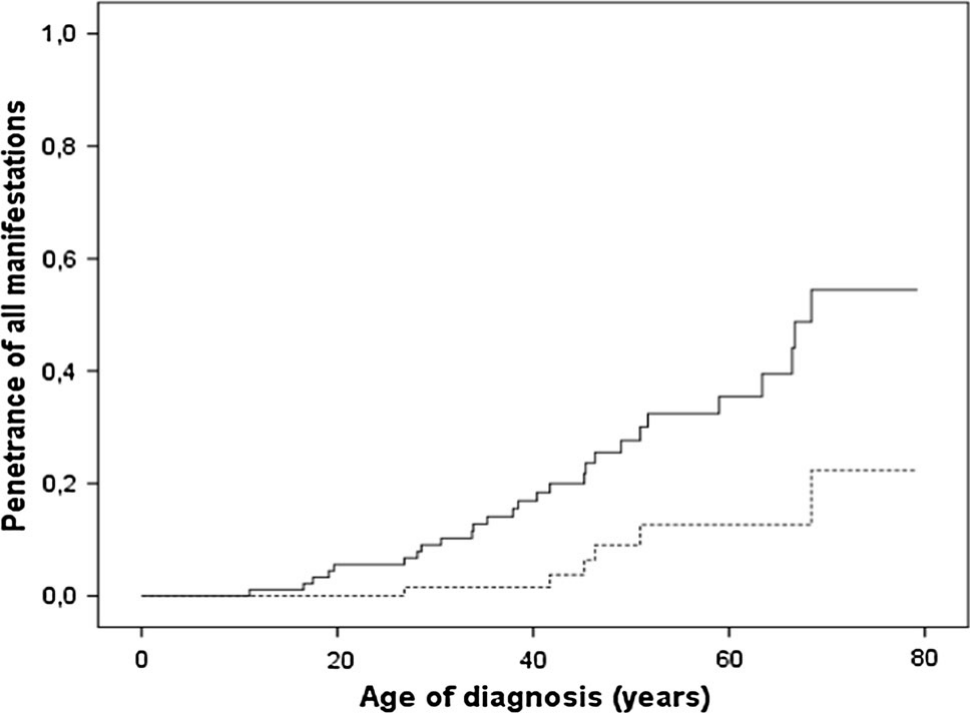
Age-related penetrance of all manifestations (head and neck paraganglioma, sympathetic paraganglioma and pheochromocytoma) of the succinate dehydrogenase subunit B-gene including index cases (*black line*) and excluding index patients (*dotted line*)

Twenty-seven *SDHB* mutation carriers (30 %) had a manifestation either at initial presentation (index patient, *n* = 21) or during follow-up (*n* = 6); overall penetrance (including the index patients) was 18 % at the age of 40 years and 35 % at the age of 60 years (Fig. 2). Overall penetrance without index patients was 2 % at age 40 and 12 % at age 60. No significant difference in penetrance was found between the three most common mutations (deletion exon 3, c.654G>A and c.653G>A) (*P* = 0.242). Because of the low number of patients, mutations in codon 649, 292, 268 and 725 were excluded from comparisons. The manifestations of each specific mutation are reported in Table 3. The median age at diagnosis of the first manifestation was 39 years [IQR 28–51, range 11–68]. The oldest clinically unaffected subject was 77 years of age.

**Table 3:** Manifestations of disease in different mutations in the *SDHB* gene

	Total (*n* = *91*)	Deletion exon 3 (*n* = *44*)	c.654G>A (*n* = 25)	c.653G>A (*n* = *14*)	c.649C>T (*n* = *1*)	c.292T>C (*n* = *4*)	c.268C>T *(n* = *1*)	c.73?−200+(*n* = *1*)	c.725G>A (*n* = 1)
HNPGL	19	11	1	5	0	1	0	0	1
Sympathetic PGL/PCC	9	5	2	1	0	0	1	0	0
Malignant PGL	8	6	1	1	0	0	0	0	0
Unaffected	64	29	22	8	1	3	0	1	0

*SDHB* succinate dehydrogenase subunit B, *HNPGL* head and neck paraganglioma, *PGL* paraganglioma, *PCC* pheochromocytoma

### Optimal surveillance

**Fig. 3:**
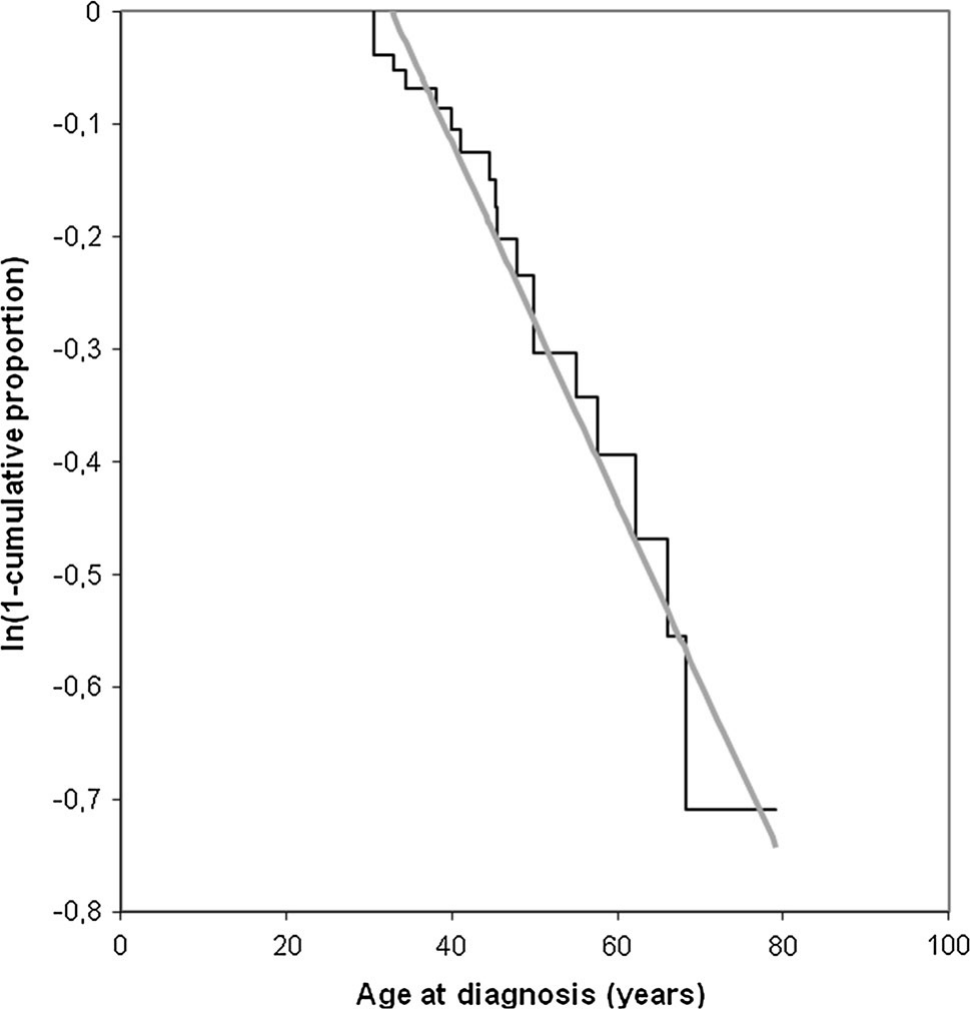
Natural logarithm of 1-cumulative proportion shown for succinate dehydrogenase subunit B mutation carriers with a head and neck paraganglioma (*black line*), corresponding to Poisson process (*grey line*)

The optimal age to start surveillance with subsequent intervals was calculated for HNPGLs. A linear correlation was found between the ln 1-cumulative proportion and the age of first HNPGL (*r* Pearson: 0.98), corresponding to the Poisson process (Fig. 3). The incidence rate (κ) of this manifestation was 1.6 % per year. The mean delay between the second hit and detection of the tumor (δ) was 32.7 years (95 % CI 27.1–38.3). As a result, the optimal age to start screening for this manifestation would be 27.1 years. Subsequent intervals were 6.6, 3.2 and 0.6 years for a detection probability of 10, 5 and 1 %, respectively. Therefore the chance to detect a HNPGL is 10, 5 or 1 % when using an interval van 6.6, 3.2 or 0.6 years respectively. The optimal surveillance for sympathetic PGLs and PCCs could not be assessed due to low number of patients.

### Other neoplasms

In addition to the patients diagnosed with a (HN)PGL or PCC, several other manifestations were reported in this cohort of *SDHB* mutation carriers. In two carriers (2 %) (one unaffected carrier and one index patient with a HNPGL) pituitary tumors were present: one non-functioning microadenoma and one microprolactinoma. Three *SDHB* mutation carriers (3 %) had a non-hypersecreting adrenal adenoma, either based on the pathology report (*n* = 1), or based on MRI and scintigraphic findings in combination with normal levels of plasma metanephrines (*n* = *2*). Renal cysts were present in six (four unaffected carriers and two index patients) carriers (7 %). Other (histologically proven) neoplasms included cervical carcinoma (*n* = 1; unaffected carrier) and melanoma (*n* = 1; index patient with HNPGL). No renal cell carcinoma, thyroid malignancy, or gastrointestinal stromal tumors was diagnosed in these carriers.

## Discussion

This is the first study using the Poisson distribution model to calculated the time to detect the first and subsequent HNPGL in *SDHB* mutation carriers. This mathematical model provides a rationale to assess the optimal age to start surveillance at 27 years and subsequent follow-up intervals at 3 years. This approach also has been recently applied to calculate the optimal surveillance in patients with a mutation in the *VHL*-gene [18]. The outcome is compatible with the assumption of the two-hit hypothesis used in our model, which is a statistical proof that the Poisson distribution model is a valid method to use. The optimal age and the interval to start screening for a sympathetic PGL/PCC could not be calculated, due to the relatively low number of patients with these manifestations in our cohort. This relatively low number of sympathetic PGL and PCC has also been described in another Dutch report about the phenotype of *SDHB* mutation carriers in the Netherlands [20], although in contrast to previous studies [6–8]. Nevertheless, the fact that four out of nine patients with a sympathetic PGL or PCC were younger than 20 years of age suggests that biochemical screening has to start at an earlier age. Of notice, no renal cell carcinomas were detected in our cohort despite a previously reported incidence of 14 % among *SDHB* mutation carriers [12].

An important question is whether our findings can be extrapolated to all *SDHB* mutation carriers, since the penetrance of manifestations in our population is somewhat lower than previously reported penetrance estimates, showing a wide range varying from 18 to 95 % at age of 60 years (Table 4) [6–8, 10–12]. This wide variation in penetrance rates is largely explained by differences between the populations studied. The three studies with the highest penetrance estimates [6, 7, 12] were mainly based on index patients, which is expected to overestimate penetrance frequency. Srirangalingam et al. [7] corrected for this bias, by excluding the index patients, which resulted in a considerably lower penetrance of 24 % (age not reported). The differences in penetrance frequencies could also be caused by variation in genotypes and, therefore, extrapolation to other genotypes might not be possible. In our study we did not find a difference between the three most frequent mutations in our study, but larger studies are needed to further explore this hypothesis.

**Table 4:** Previous studies regarding penetrance of *SDHB* mutation carriers, compared to the present cohort

	Number of *SDHB*-mutation carriers	Number of index patients	20 years	40 years	60 years	80 years	Remarks
Schiavi et al. [10]	135	n.a.	1 %	8 %	18 %	30 %	Families were excluded for which information was available for the index patient only, using a maximum likelihood method with modified segregation analysis
Ricketts et al. [12]	295	n.a.	20 %	40 %	70 %	–	Tertiary referral center
Solis et al. [11]	41	1	10 %	35 %	35 %	–	One family with a large exon-1 mutation
Srirangalingam et al. [7]	32	34 %	25 %	50 %	95 %	–	Tertiary referral center
Benn et al. [8]	82	52 %	10 %	45 %	75 %	80 %	Tertiary referral center
Neumann et al. [23]	42	60 %	30 %	55 %	95 %	100 %	Tertiary referral center
Present cohort		23 %					Tertiary referral center
Including index patients	91		6 %	18 %	35 %	49 %	
Excluding index patients	70		0 %	2 %	12 %	22 %	

*SDHB* succinate dehydrogenase subunit B, *n.a.* not applicable

Our results suggest that the screening for HNPGL in *SDHB* mutation carriers might as well start at 27 years instead of 18 years. This would result in a significant decrease in financial costs and patient burden. Although in certain families there is a request to start surveillance at an earlier age for psychological reasons, the chance to detect a HNPGL before the age of 27 years is very low (<2.5 %). As also mentioned above, we can only draw this conclusion for screening of HNPGL, screening for a sympathetic PGL or PCC probably has to start at an earlier age.

The optimal surveillance interval was set at 5 % detection probability which results in an interval of 3.2 years. This is in agreement with according to the current guidelines [3]. A detection level of 5 % is comparable to the yield of surveillance programs for breast and colon cancer [21]. HNPGLs are benign and slow growing tumors, although they can give rise to significant morbidity (i.e. cranial nerve paralysis). Given the fact that the growth rate of HNPGL is low (median doubling time of 10 years) and only a small majority of these tumors (60 %) actually grows [22], a detection level of 5 % seems justified. One limitation of this assumption is that the risk of developing metastases in patients with HNPGL is not clear from the literature. In a meta-analysis of Hulsteijn et al. [14], the risk of metastatic PGL was 17 % in patients with a *SDHB* mutation, however it is not clear how many of these were metastatic HNPGLs.

A limitation of our study is that we did not include a sufficient number of patient to draw conclusions for sympathetic PGL and PCC due to the limited penetrance of sympathetic PGL and PCC observed in the Netherlands. However, *SDHB* mutation carriers are rare and our cohort with 91 carriers was large enough to draw solid statistical conclusion about screening for HNPGL. Our findings need to be confirmed in another large population of *SDHB* mutation carriers to calculate optimal surveillance of the manifestations for HNPGL in different genotypes and for the manifestations sympathetic PGL and PCC. Another limitation is the finding that two carriers were classified as having an adrenal adenoma, although a non-functioning PCC could not be ruled out since they were not operated on.

In conclusion, we calculated the optimal surveillance for HNPGL in *SDHB* mutation carriers by using a mathematical model. Based on these data the age to start could be postponed to 27 years of age. These results may give rise to reconsider the current guidelines regarding the screening of *SDHB* mutation carriers.

## References

[1] Pillai S, Gopalan V, Smith RA (2016). Updates on the genetics and the clinical impacts on phaeochromocytoma and paraganglioma in the new era. Crit Rev Oncol Hematol.

[2] Astuti D, Latif F, Dallol A (2001). Gene mutations in the succinate dehydrogenase subunit SDHB cause susceptibility to familial pheochromocytoma and to familial paraganglioma. Am J Hum Genet.

[3] Dutch guideline for detecting hereditary tumors 2010. https://www.stoet.nl

[4] Kirmani S, Young WF (2014) Hereditary paraganglioma-pheochromocytoma syndrome. In: Pagon RA, Adam MP, Ardinger HH et al (eds) GeneReviews. University of Washington, Seattle 20301715

[5] Taieb D, Kaliski A, Boedeker CC (2014). Current approaches and recent developments in the management of head and neck paragangliomas. Endocr Rev.

[6] Neumann HP, Eng C (2009). The approach to the patient with paraganglioma. J Clin Endocrinol Metab.

[7] Srirangalingam U, Walker L, Khoo B (2008). Clinical manifestations of familial paraganglioma and phaeochromocytomas in succinate dehydrogenase B (SDH-B) gene mutation carriers. Clin Endocrinol (Oxf).

[8] Benn DE, Gimenez-Roqueplo AP, Reilly JR (2006). Clinical presentation and penetrance of pheochromocytoma/paraganglioma syndromes. J Clin Endocrinol Metab.

[9] Fishbein L, Nathanson KL (2012). Pheochromocytoma and paraganglioma: understanding the complexities of the genetic background. Cancer Genet.

[10] Schiavi F, Milne RL, Anda E (2010). Are we overestimating the penetrance of mutations in SDHB?. Hum Mutat.

[11] Solis DC, Burnichon N, Timmers HJ (2009). Penetrance and clinical consequences of a gross SDHB deletion in a large family. Clin Genet.

[12] Ricketts CJ, Forman JR, Rattenberry E (2010). Tumor risks and genotype-phenotype-proteotype analysis in 358 patients with germline mutations in SDHB and SDHD. Hum Mutat.

[13] Amar L, Baudin E, Burnichon N (2007). Succinate dehydrogenase B gene mutations predict survival in patients with malignant pheochromocytomas or paragangliomas. J Clin Endocrinol Metab.

[14] van Hulsteijn LT, Dekkers OM, Hes FJ (2012). Risk of malignant paraganglioma in SDHB-mutation and SDHD-mutation carriers: a systematic review and meta-analysis. J Med Genet.

[15] Weber A, Hoffmann MM, Neumann HP (2012). Somatic mutation analysis of the SDHB, SDHC, SDHD, and RET genes in the clinical assessment of sporadic and hereditary pheochromocytoma. Horm Cancer.

[16] Berger AH, Knudson AG, Pandolfi PP (2011). A continuum model for tumour suppression. Nature.

[17] Knudson AG (2001). Two genetic hits (more or less) to cancer. Nat Rev Cancer.

[18] Kruizinga RC, Sluiter WJ, de Vries EG (2013). Calculating optimal surveillance for detection of von Hippel–Lindau-related manifestations. Endocr Relat Cancer.

[19] de Jong WH, Graham KS, van der Molen JC (2007). Plasma free metanephrine measurement using automated online solid-phase extraction HPLC tandem mass spectrometry. Clin Chem.

[20] van Hulsteijn LT, Niemeijer ND, Hes FJ (2014). Phenotype of SDHB mutation carriers in the Netherlands. Fam Cancer.

[21] Pox CP, Altenhofen L, Brenner H (2012). Efficacy of a nationwide screening colonoscopy program for colorectal cancer. Gastroenterology.

[22] Jansen JC, van den Berg R, Kuiper A (2000). Estimation of growth rate in patients with head and neck paragangliomas influences the treatment proposal. Cancer.

[23] Neumann HPH, Pawlu C, Peczkowska M (2004). Distinct clinical features of paraganglioma syndromes associated with *SDHB* and *SDHD* gene mutations. JAMA.

